# Application of ordinal logistic regression analysis in determining risk factors of child malnutrition in Bangladesh

**DOI:** 10.1186/1475-2891-10-124

**Published:** 2011-11-14

**Authors:** Sumonkanti Das, Rajwanur M Rahman

**Affiliations:** 1 Department of Statistics, Shahjalal University of Science & Technology, Bangladesh; 2 Shafi Consultancy Bangladesh, Sylhet, Bangladesh

**Keywords:** Ordinal logistic regression model, Proportional odds model, Partial proportional odds model, Binary logistic regression model, Anthropometric index, Child malnutrition

## Abstract

**Background:**

The study attempts to develop an ordinal logistic regression (OLR) model to identify the determinants of child malnutrition instead of developing traditional binary logistic regression (BLR) model using the data of Bangladesh Demographic and Health Survey 2004.

**Methods:**

Based on weight-for-age anthropometric index (Z-score) child nutrition status is categorized into three groups-severely undernourished (< -3.0), moderately undernourished (-3.0 to -2.01) and nourished (≥-2.0). Since nutrition status is ordinal, an OLR model-proportional odds model (POM) can be developed instead of two separate BLR models to find predictors of both malnutrition and severe malnutrition if the proportional odds assumption satisfies. The assumption is satisfied with low p-value (0.144) due to violation of the assumption for one co-variate. So partial proportional odds model (PPOM) and two BLR models have also been developed to check the applicability of the OLR model. Graphical test has also been adopted for checking the proportional odds assumption.

**Results:**

All the models determine that age of child, birth interval, mothers' education, maternal nutrition, household wealth status, child feeding index, and incidence of fever, ARI & diarrhoea were the significant predictors of child malnutrition; however, results of PPOM were more precise than those of other models.

**Conclusion:**

These findings clearly justify that OLR models (POM and PPOM) are appropriate to find predictors of malnutrition instead of BLR models.

## Background

Malnutrition is one of the most important causes for improper physical and mental development of children. Child malnutrition still remains a public health problem in developing countries like Bangladesh [1,2]. It is an underlying cause of child morbidity and mortality. Two-thirds of childhood deaths occurred due to malnutrition in Bangladesh [3]. From Bangladesh Demographic and Health Survey (BDHS) 2007, it is investigated that 43% children are stunted, and 41% are underweight in Bangladesh [4]. According to WHO, these levels of stunting and underweight are above the threshold of "very high" prevalence [5]. The level of wasting (17%) also shows that children in Bangladesh were in "serious severity" [4,5]. Using BDHS 2004 data, a study observed that nearly three fifths children were malnourished-either stunted, wasted or underweight [6]. The identification of factors for child malnutrition is still the interest of many researchers. Various methods are applied to uncover the factors of child malnutrition. Among them logistic regression analysis has got most preference in previous studies [7-10]. In most of the studies, the response variable was considered as binary (nourished and undernourished); consequently the binary logistic regression model was applied in all the cases. However, the nutrition status of a child is usually classified as nourished, moderately malnourished and severely malnourished. When the researchers are interested to find the determinants of malnutrition and severe malnutrition, two separate binary logistic regression (BLR) models are required to develop by grouping the response variable into two categories [7]. This task is tedious and cumbersome due to estimation and interpretation of more parameters. However, the researcher may consider the response variable as ordinal and may apply ordinal logistic regression model for the same purpose. A few studies have been done using ordinal logistic regression model (OLR) to identify the predictors of child undernutrition [11]. In many epidemiological and medical studies, OLR model is frequently used when the response variable is ordinal in nature [12-17]. The study has made an effort to identify the predictors of child malnutrition as well as severe malnutrition for under five Bangladeshi children by developing an ordinal logistic regression model.

### Ordinal Logistic Regression Model

There are several occasions when the outcome variable is polychotomous. Such outcome variable can be classified into two categories-multinomial and ordinal. While the dependent variable is classified according to their order of magnitude, one cannot use the multinomial logistic regression model. A number of logistic regression models have been developed for analyzing ordinal response variables [12,18-24]. Moreover, when there is a need to take several factors into consideration, special multivariate analysis for ordinal data is the natural alternative. There are various approaches, such as the use of mixed models or another class of models, probit for example, but the ordinal logistic regression models have been widely used in most of the previous research works [18,19,25-33]. There are several ordinal logistic regression models such as proportional odds model (POM), two versions of the partial proportional odds model-without restrictions (PPOM-UR) and with restrictions (PPOM-R), continuous ratio model (CRM), and stereotype model (SM). The most frequently used ordinal logistic regression model in practice is the constrained cumulative logit model called the proportional odds model [18,33-35].

The POM is the most widely used in epidemiological and biomedical applications but POM leads to strong assumptions that may lead to incorrect interpretations if the assumptions are violated [28]. If the data fail to satisfy the proportional odds assumption, a valid solution is fitting a partial proportional odds model [36]. Another simple and valid approach to analyze the data is to dichotomize the ordinal response variable by means of several cut-off points and use separate binary logistic regression models for each dichotomous response variable [37]. However, Gameroff suggested that the second procedure should be avoided if possible because of the loss in statistical power and the reduced generality of the analytical solution [17].

## Methods

### Data and Variables

The study has utilized the nationwide data of BDHS 2004 where completed and plausible anthropometric data were available for 6005 (weighted) children [38]. Weight-for-age anthropometric index is an excellent overall indicator of a population's nutritional health status. Moreover, weight-for-age is a composite index of weight-for-height and height-for-age [4]. So the study considered only weight-for-age anthropometric index instead of weight-for-height and height-for-age to measure the children nutrition status. Child nutrition status was categorized into three groups-severely undernourished (< -3.0 Z-score), moderately undernourished (-3.0 to -2.01 Z-score) and nourished (≥-2.0 Z-Score). Thus nutrition status is an ordinal response variable grouped from a continuous variable.

Several socio-economic and demographic characteristics, maternal health and nutritional information, and incidence of child diseases are considered as the independent variables to develop the POM, PPOM, and separate BLR models. Mathematical forms of the models with some indication of application are shown in Table 1. Age of children, birth interval, mothers' educational status, household wealth status, child feeding status, mothers' antenatal-postnatal care status, incidence of diarrhoea, ARI, and fever are considered as the independent variables in the study. These independent variables were found significant predictors of child undernutrition in several previous studies [7-10,39-43]. Household wealth status is evaluated from household wealth index which is constructed by NIPORT *et al*. [38]. Child feeding status and mothers' antenatal-postnatal care status are evaluated by constructing child feeding index and antenatal-postnatal care index respectively. Both the indices are constructed according to previous studies by Das *et al*. [9,10]. Construction procedure is not shown in this paper.

**Table 1:** Functional form of BLR, POM and restricted and unrestricted PPOM

Model	Functional form	Indication for use
Binary Logistic Model (BLM)	$\lambda_{}\left(\underset{\to }{x}\right)=\ln\left\{\frac{\mathrm{Pr}\left(Y=1|\underset{\to }{x}\right)}{\mathrm{Pr}\left(Y=0|\underset{\to }{x}\right)}\right\}=\alpha_{}+\left(\beta_{1}x_{1}+\beta_{2}x_{2}+...+\beta_{p}x_{p}\right)$	Response variable with two categories (Y = 0,1)

Proportional Odds Model (POM)	$\begin{array}{l} \lambda_{j}\left(\underset{\to }{x}\right)=\ln\left\{\frac{\mathrm{Pr}\left(Y=1|\underset{\to }{x}\right)+...+\mathrm{Pr}\left(Y=j|\underset{\to }{x}\right)}{\mathrm{Pr}\left(Y=j+1|\underset{\to }{x}\right)+...+\mathrm{Pr}\left(Y=k|\underset{\to }{x}\right)}\right\}=\ln\left\{\frac{\overset{j}{\underset{1}{\sum }}\mathrm{Pr}\left(Y=j|\underset{\to }{x}\right)}{\overset{k}{\underset{j+1}{\sum }}\mathrm{Pr}\left(Y=j|\underset{\to }{x}\right)}\right\}\\ \lambda_{j}\left(\underset{\to }{x}\right)=\alpha_{j}+\left(\beta_{1}x_{1}+\beta_{2}x_{2}+...+\beta_{p}x_{p}\right),\text{ j}=\text{1,2,}...\text{,k - 1} \end{array}$	Originally continuous response variable, subsequently grouped, and valid proportional odds assumption

Unrestricted Partial Proportional Odds Model (PPOM-UR)*	$\begin{array}{l} \lambda_{j}\left(\underset{\to }{x}\right)=\ln\left\{\frac{\mathrm{Pr}\left(Y=1|\underset{\to }{x}\right)+...+\mathrm{Pr}\left(Y=j|\underset{\to }{x}\right)}{\mathrm{Pr}\left(Y=j+1|\underset{\to }{x}\right)+...+\mathrm{Pr}\left(Y=k|\underset{\to }{x}\right)}\right\}=\ln\left\{\frac{\overset{j}{\underset{1}{\sum }}\mathrm{Pr}\left(Y=j|\underset{\to }{x}\right)}{\overset{k}{\underset{j+1}{\sum }}\mathrm{Pr}\left(Y=j|\underset{\to }{x}\right)}\right\}\\ \lambda_{j}\left(\underset{\to }{x}\right)=\alpha_{j}+\left[\left(\beta_{1}+\gamma_{j1}\right)x_{1}+...+\left(\beta_{q}+\gamma_{jq}\right)x_{q}+\left(\beta_{q+1}\right)x_{q+1}...+\beta_{p}x_{p}\right],\text{ j}=\text{1,2,}...\text{,k - 1} \end{array}$	Proportional odds assumption not valid

Restricted Partial Proportional Odds Model (PPOM-R)	$\begin{array}{l} \lambda_{j}\left(\underset{\to }{x}\right)=\ln\left\{\frac{\mathrm{Pr}\left(Y=1|\underset{\to }{x}\right)+...+\mathrm{Pr}\left(Y=j|\underset{\to }{x}\right)}{\mathrm{Pr}\left(Y=j+1|\underset{\to }{x}\right)+...+\mathrm{Pr}\left(Y=k|\underset{\to }{x}\right)}\right\}=\ln\left\{\frac{\overset{j}{\underset{1}{\sum }}\mathrm{Pr}\left(Y=j|\underset{\to }{x}\right)}{\overset{k}{\underset{j+1}{\sum }}\mathrm{Pr}\left(Y=j|\underset{\to }{x}\right)}\right\}\\ \lambda_{j}\left(\underset{\to }{x}\right)=\alpha_{j}+\left[\tau_{j}\left\{\left(\beta_{1}+\gamma_{j1}\right)x_{1}+...+\left(\beta_{q}+\gamma_{jq}\right)x_{q}\right\}+\left(\beta_{q+1}\right)x_{q+1}...+\beta_{p}x_{p}\right],\text{ j}=\text{1,2,}...\text{,k - 1} \end{array}$	Proportional odds assumption not valid, and linear relationship for odds ratio (OR) between a co-variable and the response variable

Note: Y = Response variable, $\underset{\to }{x}$ vector of explanatory variables = (*x*_1_, *x*_2_, ....., *x_p_*)

*Stata uses P > j vs. < = j for the probability comparison in case of PPOM.

The authority of DHS maintains all kinds of ethical standards and procedures for the survey and also takes informed consent from the survey respondents before the data collection. In addition, we have obtained approval from the DHS to use the data through the website of DHS. So no ethical approval is needed for the study from any other institutions.

### Model Fitting

Since the response variable "nutrition status" is ordinal in nature (grouped from continuous variable-weight-for-age anthropometric index), at first POM was formed without a careful assessment of the model adequacy. The chi-squared score test for the proportional odds assumption [18,36] was employed to see whether the main model assumption was violated or not. As the score test is often anticonservative (i.e., the resulting P-values are far too small) [13,24,36], we use other techniques to investigate the proportional odds assumption. We calculated single score tests for each covariate for checking whether proportional odds assumption is violated [24]. Graphical method has also been employed for checking the parallel slope assumptions for all co-variables. In addition, separate binary logistic regression analyses have been conducted as a basis for more careful analysis [26]. We dichotomized the response variable taking account of the ordering by using cumulative probabilities. The response variable is dichotomized as "at least moderate undernutrition" with two categories '0' = no undernutrition & '1' = at least moderate undernutrition and "at least severe undernutrition" with two categories '0' = no undernutrition or moderate undernutrition and '1' = at least severe undernutrition. The overall goodness-of-fit of the separate BLR models was assessed by "Hosmer and Lemeshow test" [33,44,45].

Though POM is suitable for analyzing ordinal variables arising from a continuous variable, the proportional odds assumption is satisfied seldom in practice. When this assumption is violated, a legal alternative is to develop a PPOM which allows some co-variables with proportional odds assumption to be modeled, but for the co-variables failed to perform the proportional odds assumption, it is augmented by a coefficient (γ), which is the effect linked with each j-th cumulative logit, adjusted by the other co-variables [33]. Thus, PPOM releases the constraint of having a common parameter across the response logits for all the predictors considered in the model [17]. Since both PPOM and separate binary logistic regression approaches are based on cumulative logit, the PPOM is directly comparable with separate BLR models [37]. In the same way, the formulation of the logit functions in POM and PPOM are identical (i.e. nourish vs. moderately & severely undernourish; nourish and moderately undernourish vs. severely undernourish), so overall fit of these two models are comparable [28]. So the study compared the results of the separate BLR models with that of the PPOM, and also compared POM with PPOM. The study fitted unrestricted PPOM model. STATA procedure OLOGIT and SPSS procedure PLUM with TPARALLEL option for POM, SPSS procedure LOGISTIC REGRESSION for separate BLR models [46], STATA procedure GOLOGIT2 with AUTOFIT option for PPOM [47] were employed in the study. For graphical tests of proportional odds assumption, PROC LOGISTIC procedure of SAS is used to obtain the estimated logits for at least moderate under-nutrition (logit {P[Y≤1]/P[Y = 2]}) and for at least severe undernutrition (logit {P[Y = 0]/P[Y≥1]}). To see the parallel regression assumptions, estimated logits are plotted against all categories of each explanatory variable. SPSS 17.0, STATA 11.1, and SAS 9.2 are utilized for the complex statistical analysis.

## Results

The proportion of undernourished children was 48% with 13% severely undernourished in 2004. Though both the levels reduced in 2007 (43% with 12% severely underweight), the levels are still very high [4]. The prevalence of child malnutrition according to selected background characteristics are shown in Table 2. The proportion of severely malnourished and moderately malnourished children were found higher among the children aged 12-23 months (18% & 41%), having < 24 months birth interval (13% & 35%), illiterate (17% & 38%) and acutely malnourished mothers (18% & 40%), worst child feeding status (16% & 36%), and experienced with several diseases like diarrhea, ARI, and fever (near about 50% malnourished separately). Moreover, near about three-fourth children lived in poorest households were most vulnerable to malnutrition. All the selected independent variables were significantly associated with the children's malnutrition status (Chi-square statistics and p-values are mentioned in Table 2).

**Table 2:** Children's nutrition status according to selected independent variables

Co-variables	Nutrition Status according to Weight-for-Age Z-score (WAZ)				Pearson Chi-square (p-value)
		
	Severe Malnourish (WAZ < -3.00)	Moderate Malnourish (-3.00≤WAZ≤2.01)	Nourish (WAZ≥-2.00)	Total	
**Children age (in months)**					

0-11	5.2	14.4	80.5	1140	455.986 (0.000)
12-23	17.8	41.0	41.2	1170	
24^+^	13.6	38.6	47.7	3695	

**Birth interval (months)**					

48+ months	10.6	31.0	58.4	1787	36.924 (0.000)
24-47 months	14.3	36.4	49.3	2586	
< 24 months	13.0	35.2	51.8	1523	

**Mother's education**					

Higher	3.4	17.4	79.1	422	204.092 (0.000)
Secondary	10.4	33.1	56.5	1987	
Primary	12.6	35.9	51.5	1292	
No education	17.1	38.1	44.8	2217	

**Household wealth status**					

Richest	5.9	24.3	69.7	987	251.337 (0.000)
Richer	10.5	32.1	57.4	1091	
Middle	12.0	32.5	55.6	1179	
Poor	14.4	38.5	47.1	1237	
Poorest	18.4	41.2	40.3	1512	

**Child feeding status**					

High (10-12)	8.5	26.0	65.5	1148	99.780 (0.000)
Medium (7-9)	13.2	36.8	50.0	3367	
Low (0-6)	15.5	35.7	48.7	1381	

**Mothers' antenatal-postnatal care status**					

Sufficient (16-18)	3.6	17.5	78.8	290	136.482 (0.000)
Merely sufficient (11-15)	10.1	27.1	62.9	796	
Less sufficient (6-10)	11.3	33.0	55.8	1396	
Least sufficient (1-5)	13.8	36.1	50.1	1102	
No care (0)	16.5	36.8	46.7	1137	

**Mother's BMI**					

Normal (≥18.5)	9.5	31.0	59.4	3664	198.723 (0.000)
Thinness (< 18.5)	18.3	40.1	41.7	2232	

**Incidence of ARI in the last two weeks**					

No	12.2	33.8	54.0	4654	15.833 (0.000)
Yes	15.1	36.9	48.0	1242	

**Incidence of fever in the last two weeks**					

No	11.2	33.3	55.5	3499	34.490 (0.000)
Yes	15.2	36.1	48.6	2397	

**Incidence of diarrhoea in the last two weeks**					

No	12.5	34.2	53.3	5445	13.350 (0.001)
Yes	16.9	38.0	45.1	451	

**Total**	12.8	34.5	52.7	6005	N/A

To identify the risk factors of child malnutrition, the study fitted POM, separate BLR models, and PPOM. At first competence of the models are described and then the results of the models are interpreted.

### Proportional Odds Model

The results of the multiple POM are given in Table 3. All the considered variables in the POM are found significant. The score test of the proportional odds assumption is found insignificant at 5% level of significance indicating the data satisfy the proportional odds assumption. However, the p-value of the score test is found small (0.144). To confirm the conclusion regarding the assumption of POM, single score tests of the proportional odds assumption for each covariate were conducted. The p-values of the single score tests are shown in the last column of Table 3. The test results reveal that all the variables except age of children (p-value < 0.005) were found insignificant i.e., satisfy the proportional odds assumption. Without making a final decision we proceed to analyze the data using separate binary logistic regressions for the dichotomized response. Such an analysis is required to assess the correct functional form of the covariates to build models with adequate goodness-of-fit.

**Table 3:** Results of the multiple POM using nutrition status as response three ordered categories ^♣^

Co-variable	Regression coefficient	Standard error	p-value	Odds ration	95% CI of OR	Single score test (p-value)
**Intercept _1_**	3.888	.209	.000	**-**	**-**	**-**
	
**Intercept _2_**	5.888	.218	.000	**-**	**-**	

**Children age (in months) [0-11 months as Reference]**						

12-23	1.877	.099	.000	6.534	5.381-7.934	**0.005**
24^+^	1.638	.091	.000	5.147	4.302-6.157	

**Birth interval (months) [48+ months as reference]**						

24-47	.403	.071	.000	1.496	1.301-1.719	**0.928**
< 24	.465	.083	.000	1.591	1.353-1.871	

**Mother's education [Higher education as reference]**						

Secondary	.820	.156	.000	2.270	1.672-3.080	**0.549**
Primary	.760	.167	.000	2.139	1.542-2.967	
No education	.982	.166	.000	2.670	1.929-3.694	

**Household wealth status [Richest as reference]**						

Richer	.273	.112	.015	1.314	1.054-1.638	**0.799**
Middle	.359	.115	.002	1.432	1.144-1.792	
Poorer	.534	.116	.000	1.705	1.359-2.139	
Poorest	.695	.118	.000	2.005	1.590-2.527	

**Child feeding status [High (10-12) as reference]**						

Medium (7-9)	.045	.081	.578	1.046	0.893-1.226	**0.180**
Low (0-6)	.248	.095	.009	1.281	1.063-1.544	

**Mothers' antenatal-postnatal care status [Sufficient (16-18) as reference]**						

Merely sufficient (11-15)	.338	.177	.057	1.402	0.990-1.984	**0.678**
Less sufficient (6-10)	.318	.177	.073	1.375	0.971-1.946	
Least sufficient (1-5)	.453	.182	.013	1.573	1.101-2.246	
No care (0)	.472	.185	.011	1.603	1.116-2.301	

**Mother's BMI [**Normal (≥18.5) as reference]						

Thinness (< 18.5)	.553	.063	.000	1.738	1.537-1.965	**0.665**

**Incidence of ARI in the last two weeks [No as reference]**						

Yes	.195	.078	.012	1.215	1.044-1.415	**0.678**

**Incidence of fever in the last two weeks [No as reference]**						

Yes	.234	.066	.000	1.263	1.111-1.436	**0.292**

**Incidence of diarrhoea in the last two weeks [No as reference]**						

Yes	.245	.107	.021	1.278	1.037-1.575	**0.876**

**Score test for the proportional odds assumption: **Chi-square = 27.83, df = 21, p-value = 0.144						
**Goodness-of-fit test of overall model (Likelihood Ratio): **Chi-square = 926.52, df = 21, p-value = 0.000, Pseudo R^2 ^= 0.1029						

^♣ ^Sample Size: 4720

### Separate Binary Logistic Regressions

Results of two separate binary logistic regression models are shown in Table 4. Hosmer-Lemeshow test for both the models indicate that both the models have no lack of fit (p-value > 0.58). The regression coefficients and odds ratios in the two separate models for all the categories of each of the covariates are found homogeneous. The age of children which fails to satisfy the proportional odds assumption has the significant influence on both the models. However, significance levels varied for some covariates in the two BLR models. In the first BLR model with response variable "at least moderate undernutrition" all the variables are found significant. On the other hand, mothers' antenatal-postnatal care, incidence of ARI and diarrhea are found insignificant in the other BLR model with response variable "at least severe undernutrition". Thus the covariates show satisfactory result with some differences in significance level. Since these regression models do not consider the restriction of ordinal response and consider more parameters, we proceed to construct PPOM, which represents a joint model of the response categories [22], a powerful method based upon maximum likelihood procedures for ordinal response [23].

**Table 4:** Results of two separate multiple binary logistic regression models using child nutrition status as binary response ^♣^

	Comparisons					
	
Co-variable	Nourish vs. (moderately & severely malnourished) ^1^			Nourish & moderately malnourished vs. (severely malnourished) ^2^		
	
	β_1_	OR_1_	CI (P-value)	β_2_	OR_2_	CI (P-value)
Coefficient	-3.976	-	.000			

**Children age (in months) [0-11 months as Reference]**						

12-23	1.943	6.977	5.69-8.55 (.000)	1.445	4.242	3.08-5.84 (.000)
24^+^	1.683	5.381	4.47-6.48 (.000)	1.207	3.343	2.46-4.55 (.000)

**Birth interval (months) [48+ months as reference]**						

24-47	.433	1.542	1.33-1.79 (.000)	.284	1.329	1.07-1.65 (.009)
< 24	.514	1.671	1.41-1.99 (.000)	.316	1.371	1.07-1.76 (.014)

**Mother's education [Higher education as reference]**						

Secondary	.827	2.286	1.68-3.12 (.000)	.823	2.276	1.22-4.25 (.010)
Primary	.776	2.172	1.55-3.04 (.000)	.797	2.219	1.16-4.24 (.016)
No education	.954	2.596	1.86-3.62 (.000)	1.141	3.131	1.65-5.94 (.000)

**Household wealth status [Richest as reference]**						

Richer	.260	1.297	1.03-1.63 (.025)	.338	1.402	0.95-2.07 (.088)
Middle	.352	1.421	1.13-1.80 (.003)	.438	1.549	1.05-2.28 (.027)
Poorer	.594	1.812	1.43-2.30 (.000)	.426	1.531	1.04-2.26 (.032)
Poorest	.698	2.011	1.58-2.57 (.000)	.687	1.988	1.35-2.93 (.000)

**Child feeding status [High (10-12) as reference]**						

Medium (7-9)	.072	1.075	0.91-1.27 (.398)	.005	1.005	0.78-1.30 (.970)
Low (0-6)	.240	1.271	1.04-1.55 (.019)	.305	1.357	1.02-1.81 (.038)

**Mothers' antenatal-postnatal care status [Sufficient (16-18) as reference]**						

Merely sufficient (11-15)	.305	1.357	0.95-1.94 (.092)	.510	1.665	0.83-3.34 (.150)
Less sufficient (6-10)	.330	1.390	0.98-1.98 (.069)	.339	1.403	0.70-2.81 (.339)
Least sufficient (1-5)	.462	1.587	1.10-2.29 (.013)	.459	1.582	0.78-3.19 (.200)
No care (0)	.455	1.576	1.09-2.29 (.017)	.510	1.665	0.82-3.37 (.157)

**Mother's BMI [**Normal (≥18.5) as reference]						

Thinness (< 18.5)	.581	1.789	1.57-2.04 (.000)	.541	1.717	1.43-2.06 (.000)

**Incidence of ARI in the last two weeks [No as reference]**						

Yes	.242	1.274	1.08-1.50 (.004)	.117	1.124	0.90-1.41 (.308)

**Incidence of fever in the last two weeks [No as reference]**						

Yes	.211	1.234	1.08-1.42 (.003)	.286	1.331	1.10-1.62 (.004)

**Incidence of diarrhoea in the last two weeks [No as reference]**						

Yes	.271	1.311	1.04-1.65 (.023)	.150	1.161	0.86-1.57 (.327)

**Hosmer-Lemeshow goodness-of-fit test**	**p-value = 0.589**			**p-value = 0.610**		

^♣ ^Sample size: 4745

### Partial Proportional Odds Model

The results of default GOLOGIT2 of STATA are similar to the series of binary logistic regressions and can be interpreted in the same way. The main problem with the results of both processes is that they include many more parameters than POM. These methods free all the variables from the parallel-lines constraint, even though the assumption may be violated only by one or a few of them. So the study used AUTOFIT option with GOLOGIT2 to fit partial proportional odds models, where the parallel-lines constraint is relaxed only for those variables where the assumption was not justified and parallel-lines constraint is considered for the other variables which satisfy the assumption [46]. The results are shown in Table 5 with Wald test of parallel-lines assumption. Global Wald test for the final model indicates that final model does not violate the proportional odds assumption with high p-value: 0.7943. From Table 4 and Table 5 it is clear that only 23 unique β coefficients or odds ratios need to be explained in PPOM compared to the 42 coefficients produced by separate BLR models. Results of PPOM show that all the covariates have significant influence on the response variable in both comparisons. In addition, the deviance (defined as the difference in the likelihood ratios between POM and PPOM) is chi-square = 15.03 (941.55-926.52) with 2 d.f. (23-21), favouring the PPOM as a better fit to the data than POM [28]. The pseudo R^2 ^of POM (0.1029) and PPOM (0.1046) also reflect the same result.

**Table 5:** Results of multiple PPOM using child nutrition status as response with three ordered categories ^♣^

	Comparisons					
**Co-variable**	**Nourish vs. (moderately & severely malnourished)**			**Nourish & moderately malnourished vs. (severely malnourished)**		
	
	**β_1_**	**OR_1_**	**P-value**	**β_2_**	**OR_2_**	**P-value**

Coefficient	3.9269	-	0.000	5.576	-	0.000

**Children age (in months) [0-11 months as Reference]**						

12-23	1.9264	6.8645	0.000	1.4328	4.1904	0.000
24-35	1.6772	5.3508	0.000	1.1986	3.3156	0.000

**Birth interval (months) [48+ months as reference]**						

24-47	0.4035	1.4971	0.000	0.4035	1.4971	0.000
< 24	0.4683	1.5973	0.000	0.4683	1.5973	0.000

**Mother's education [Higher education as reference]**						

Secondary	0.8323	2.2987	0.000	0.8323	2.2987	0.000
Primary	0.7759	2.1726	0.000	0.7759	2.1726	0.000
No education	0. 9467	2.5772	0.000	0. 9467	2.5772	0.000

**Household wealth status [Richest as reference]**						

Richer	0.2786	1.3213	0.014	0.2786	1.3213	0.014
Middle	0.368	1.4449	0.001	0.368	1.4449	0.001
Poorer	0.5437	1.7224	0.000	0.5437	1.7224	0.000
Poorest	0.7068	2.0276	0.000	0.7068	2.0276	0.000

**Child feeding status [High as reference]**						

Medium	0.0407	1.0415	0.617	0.0407	1.0415	0.617
Low	0.2424	1.2744	0.012	0.2424	1.2744	0.012

**Mothers' antenatal-postnatal care status [Sufficient as reference]**						

Merely sufficient	0.3418	1.4075	0.055	0.3418	1.4075	0.055
Less sufficient	0.3238	1.3823	0.068	0.3238	1.3823	0.068
Least sufficient	0.4588	1.5822	0.012	0.4588	1.5822	0.012
No care	0.4796	1.6155	0.009	0.4796	1.6155	0.009

**Mother's BMI [**Normal (≥18.5) as reference]						

Thinness (< 18.5)	0.5557	1.7432	0.000	0.5557	1.7432	0.000

**Incidence of ARI in the last two weeks [No as reference]**						

Yes	0.1961	1.2167	0.011	0.1961	1.2167	0.011

**Incidence of fever in the last two weeks [No as reference]**						

Yes	0.2363	1.2665	0.000	0.243	1.2751	0.022

**Incidence of diarrhoea in the last two weeks [No as reference]**						

Yes	0.2430	1.2751	0.022	0.2363	1.2665	0.000

**Score test for the proportional odds assumption**: Chi-square = 12.95, df = 19, p-value = 0.7943						
**Goodness-of-fit test of overall model (Likelihood Ratio): **Chi-square = 941.55, df = 23, p-value = 0.000, Pseudo R^2 ^= 0.1046						

^♣ ^Sample size: 4745

### Graphical Test of Proportional Odds Assumption

**Figure 1:**
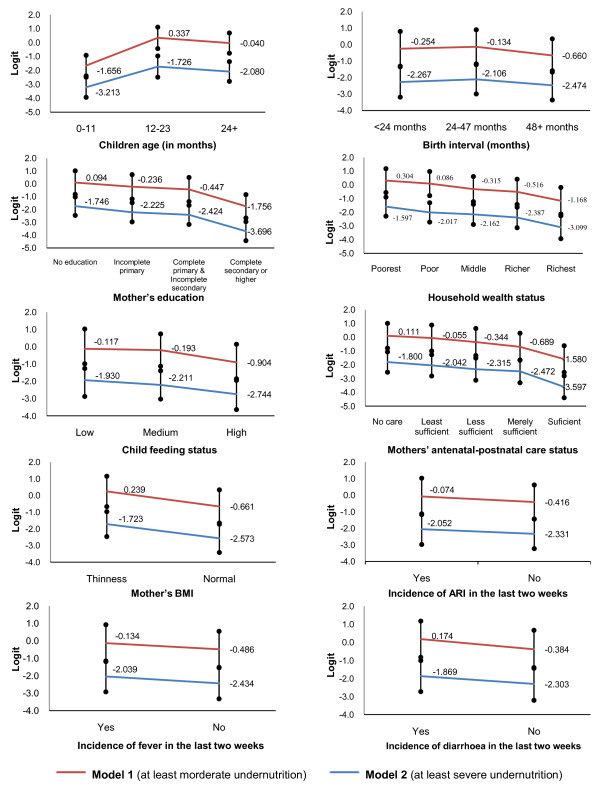
**Graphical test for proportional odds assumption**. *Model 1 *indicates the BLR model considering the response variable dichotomized as '0' = no undernutrition & '1' = at least moderate undernutrition. *Model 2 *indicates the BLR model considering response variable dichotomized as '0' = no undernutrition or moderate undernutrition and '1' = at least severe undernutrition.

The line diagrams of all the explanatory variables are shown in Figure 1. The graphical test of proportional odds assumption indicates that the estimated average logits for all categories in the distinct variable are almost parallel in shape except the variable "age of children". The average logits of different categories for the children age did not support the parallel assumption of POM. This picture is also revealed by the individual score test.

### Determinants of Child Undernutrition

In POM and PPOM, all the considered variables are found as significant predictors of child malnutrition as in previous studies. The covariates were also found significant in both the separate BLR models except antenatal-postnatal care status, incidence of diarrhoea and ARI in the 2^nd ^BLR model with the response variable "at least severe undernutrition". These results support the use of POM and PPOM instead of BLR models to determine the predictors of child undernutrition as well as severe undernutrition.

The results of POM reveal that the risk of having worse nutrition status were 6.53 and 5.15 times higher among the children belonging to the age group 12-23 and 24+ months respectively, when compared with the infants (Table 3). Since this variable violated the proportional odds assumption, this interpretation may be invalid. However, from separate BLR models and PPOM it is clear that the odds ratios for the children aged 12-23 months and 24^+ ^months compared to infants were about 6.9 and 5.4 respectively when no undernutrition state is compared with moderate and severe undernutrition states (Table 4 &5). When no undernutrition and moderate undernutrition states are compared with severe undernutrition state, the odds ratios were found about 4.2 and 3.3 respectively for children belonging to age group 12-23 and 24^+ ^months compared to infants (Table 4 &5). Since all other covariates did not violate the proportional odds assumption and PPOM performed better than POM as well as separate BLR models, the results for other covariates are described from Table 5.

Children having birth interval < 24 and 24-47 months had 1.6 and 1.5 times greater risk of having worse nutrition status compared with the children having 48^+ ^months birth interval (Table 5). The risk of having worse nutrition status was found highest for the children having mothers with no education (about 3.0 times) when compared with highly educated mothers' children. Compared to the children of the richest households, the chances of having worse nutrition status was found to increase with decrease of household wealth condition (2.03 for the children of poorest household and 1.32 for those of richer household). The risk of having poor nutrition condition was found significantly higher for the children with poor feeding practices compared to those having better feeding practices. Mothers who received no antenatal-postnatal care had 1.62 times greater risk of having malnourished children compared to those received sufficient care. The risk reduced with the increase of mothers' antenatal-postnatal care. Children belonging to acutely malnourished mothers, compared to those of nourished mothers, were 1.74 times (95% CI: 1.54-1.97) as likely to be malnourished moderately or severely. Table 5 also shows that children of acutely malnourished mothers had 1.74 times greater risk of being undernourished compared to those of nourished mothers. Children experienced with ARI, fever, and diarrhoea within last two weeks of the survey had 1.22, 1.27 and 1.28 times higher risk of being undernourished respectively when comparison is made with the children having no such problems (Table 5).

## Discussion

At first sight the POM seems to be an appropriate model for analyzing the considered data since the p-value of chi-squared score test for overall model is insignificant at 5% level of significance indicating proportional odds assumption is not violated. All of the considered variables were found significant in the POM. However, the p-value of the score test for overall model was very much small which compels to conduct single score test for each covariate. These tests show that only 'age of children' violates the vital assumption of POM which may lead invalid results. Separate BLR models also indicate the coefficients and the odds ratios for the each age categories varied in the models. Graphical test of proportional odds assumption reveals the same result. In case of all other variables, coefficients and odds ratios are not identical but almost closer. In PPOM, coefficients and odds ratios for the variable 'age of children' are almost same with the result of BLR models. However, the coefficients and odds ratios for other covariates in PPOM are slightly different compared to separate binary logistic regression models, but almost identical with those of POM. Moreover, all the variables are significant in PPOM but in separate binary logistic regression models few are insignificant.

## Conclusion

Despite some differences in the results of the fitted models, the results of POM and PPOM are reasonably comparable with those of BLR models. The POM and PPOM have proved adequate for data analysis of child nutritional status, due to the nature of the response variable (grouped continuous variable), in addition, the parsimony and ease of interpretation. Furthermore, PPOM is fitted better for the data than POM. From the results of POM and PPOM it is clear that all the considered variables in the study are significant predictors of child malnutrition as previous research works. Moreover, these findings clearly justify that OLR models (POM & PPOM) are appropriate to find predictors of malnutrition as well as sever undernutrition instead of using two separate binary logistic regression models.

## List of Abbreviations

**ARI**: Acute Respiratory Infections; **BDHS**: Bangladesh Demographic and Health Survey; **BLR**: Binary Logistic Regression; **BMI**: Body Mass Index; **CI**: Confidence Interval; **CRM**: Continuous Ratio Model; **NIPORT**: National Institute of Population Research and Training; **OLR**: Ordinal Logistic Regression; **OR**: Odds Ratio; **POM**: Proportional Odds Model; **PPOM**: Partial Proportional Odds Model; **PPOM-R**: Partial Proportional Odds Model-With Restrictions; **PPOM-UR**: Partial Proportional Odds Model-Without Restrictions; **SM**: Stereotype Model; **WAZ**: Weight-for-Age Z-score; **WHO**: World Health Organization.

## Competing interests

The authors declare that they have no competing interests.

## Authors' contributions

SD determined the study design, performed the statistical analysis, interpreted the data and drafted the typescript. RMR performed the statistical data analysis and critically reviewed the typescript. Both authors reviewed and approved the final version submitted for publication.

## Author's information

**SD**, Faculty, Department of Statistics, Shahjalal University of Science & Technology (SUST), Sylhet, Bangladesh, obtained his graduation and M.S. in Statistics from the same university. His research and publications focus on the following areas: Time Series Analysis, Stochastic Model Building, Bio-Statistics, Child Health & Nutrition, and Analytic Hierarchy Process. He carried out his M.S. Thesis on Child Health and Nutrition.

**RMR **is working as Statistical Programmer in Shafi Consultancy Bangladesh, Sylhet, Bangladesh. He completed his graduation and M.S. in Statistics from Department of Statistics, SUST, Sylhet, Bangladesh. His Interested areas of research are Data Management, Bio-statistics, Time Series Modeling, and Child Health & Nutrition.
